# Oral status is associated with chewing difficulty in Thai older adults: data from a National Oral Health Survey

**DOI:** 10.1186/s12903-023-02742-4

**Published:** 2023-01-22

**Authors:** Issarapong Kaewkamnerdpong, Punkanit Harirugsakul, Piyada Prasertsom, Warangkana Vejvithee, Kornkamol Niyomsilp, Orachad Gururatana

**Affiliations:** 1 Department of Community Dentistry, Faculty of Dentistry, Chulalongkorn University, Bangkok, Thailand; 2 Department of Oral Diagnosis and Oral Surgery, Faculty of Dentistry, Western University, Pathumthani, Thailand; 3 Department of Health, Bureau of Dental Health, Ministry of Public Health, Nonthaburi, Thailand; 4 Sirindhorn College of Public Health Chonburi, Faculty of Public Health and Allied Health Sciences, Praboromarajchanok Institute, Ministry of Public Health, 29 Wachiraprakarn Road, Chonburi Province, Postcode, 20000 Thailand

**Keywords:** Oral status, Chewing difficulty, Older adults, Thailand

## Abstract

**Background:**

The number of older adults in Thailand is increasing. Better chewing ability is associated with healthy aging. Although numerous studies have demonstrated the relationship between social backgrounds, dental service utilization, oral status and chewing difficulty, there is no study in Thailand using national oral health data to identify the variables involved with chewing difficulty among Thai older adults. Therefore, the aim of this study was to determine the association between oral status, and chewing difficulty, adjusting for social backgrounds, and dental service utilization among Thai older adults.

**Methods:**

This cross-sectional study used data from the eighth Thai National Oral Health Survey (TNOHS). A stratified multi-stage method was used for sample selection. The eighth TNOHS was conducted from June–August 2017. Data were collected using interviews and clinical oral examinations by trained interviewers and trained dentists, respectively. The bivariate analysis, chi-square test was used to explore the associations between social backgrounds, dental service utilization, oral status, and chewing difficulty. Dependent variables with *p*-values of < 0.2 for their association with independent variables in the bivariate analysis were entered into the multiple logistic regression models.

**Results:**

This study found that older adults with at least 27 teeth (*p* < 0.05), or at least eight occlusal pairs (*p* < 0.05) or income exceeding 15,000 baht per month (*p* < 0.05) were more likely to have less chewing difficulty (*p* < 0.001), while the elderly who utilized dental services in the past 12 months were associated with more chewing difficulty than those who did not utilize dental services in the past 12 months (*p* < 0.001).

**Conclusions:**

We suggest that policymakers increase the number of preventive plans and set a goal for more than 20 remaining natural teeth and four posterior occlusal pairs in young and working aged people, especially in the low income group.

## Background

The current number of older adults in Thailand has increased rapidly and will continue to do so in future decades. By 2040, Thailand’s aging population is expected to be approximately 17 million, which is more than 25% of the population [1]. The Ministry of Public Health of Thailand has proposed a goal for older adults to have at least 20 natural teeth and four posterior occlusal pairs [2]. The World Health Organization (WHO) has recommended that older people have at least 20 natural teeth [3], and the World Dental Federation (FDI) has suggested that older adults aged 65 years and above should have 21 or more teeth [4]. Data from the seventh Thai National Oral Health Survey (TNOHS) showed that 60.6% of adults between 60 and 74 years old had chewing difficulty [5]. Chewing is an important step in the digestion process, and chewing ability depends on the teeth in the maxilla and mandible [6]. It is also related to occlusion, the periodontium, temporomandibular joints (TMJs), masticatory muscles, palate and salivary glands [7]. Better chewing ability is associated with better oral health related quality of life (OHRQoL), general health-related quality of life and well-being. Therefore, chewing ability is beneficial to healthy aging [8, 9]. Chewing ability influences cognitive functioning, so older adults with better chewing ability have better cognitive functioning, activities of daily living (ADLs) and nutritional status [8, 10].

Factors associated with chewing difficulty have been reported. Tooth loss is associated with eating and chewing difficulty in older adults [9, 11]. Another study also reported that the number of teeth and occluding pairs of posterior teeth were strongly associated with ability to eat certain foods [12]. Tooth loss may lead to avoidance of certain types of food, loss of appetite, and malnutrition [13, 14]. Dry mouth measured by low moisture of the oral mucosa is associated with poor chewing [15]. In addition, dry mouth can affect the comfort of eating [16]. There is a relationship between prosthesis status and chewing difficulty. Use of unadjusted prosthesis is associated with chewing difficulty [11]. Older adults from poor social backgrounds with older age [11, 15], lower income and lower education have more difficulty chewing than their counterparts [17]. Older adults in Thailand are entitled to the Civil Servant Medical Benefit Scheme (CSMBS), the Universal Coverage Scheme (UCS) and the Social Security Scheme (SSS). Each scheme provides different free dental care coverage at government health facilities. Most of the schemes cover restorations, periodontal treatment, extractions, and acrylic-based dentures. In addition, endodontic and fixed prosthesis (crown and bridge) treatments are included only in the CSMBS, but with a limited rate of reimbursement [18]. Several studies have shown that dental service utilization (DSU) is associated with dental problems. A study from Canada revealed that immigrants who had dental problems were more likely to increase their use of dental services [19]. Another study in Sudan found that DSU in the 12 months preceding the study, or more than 12 months, was related to chewing difficulty [20]. A study from New Zealand demonstrated that non-regular dental attendees were related to higher Oral Health Impact Profile (OHIP) scores, indicating more impact and lower self-rated oral health scores [21].

Although numerous studies have demonstrated the relationship between social backgrounds, DSU, oral status and chewing difficulty, to the best of our knowledge, there have been no studies in Thailand using national oral health data to identify which social backgrounds, DSU or oral statuses are associated with chewing difficulty among Thai older adults. The aim of this study was to determine the association between oral status, and chewing difficulty adjusting for social backgrounds, and DSU among Thai older adults. In this study, the research question was aimed at exploring the factors associated with chewing difficulty by using data from the eighth Thai National Oral Health Survey (TNOHS). Lessons from Thailand’s experience might be beneficial to other developing countries.

## Methods

This cross-sectional study used data from the eighth TNOHS. A stratified multi-stage method was used for sample selection. Geographically, the country is divided into five strata comprising Bangkok and four regions (north, south, central and northeast). The four regions are divided into 12 health sectors. One health sector consists of two provinces and one province consists of four districts, except for Bangkok. For Bangkok, six sub-districts were randomly selected. Samples within each selected area were randomly drawn from the citizen registry. The sample size within each selected area was based on the proportion of municipal and rural population proportions calculated by using the dental caries prevalence in each age group obtained from the seventh TNOHS, a relative d of 10–15%, a 95% confidence interval and a design effect of 2 [22].

The sample size of our study was calculated by using the proportions of older adults with oral impact who had 11–19 missing teeth (0.64) and the proportion of older adults with oral impact who had at least 20 missing teeth (0.685) [23], using 80% power and a 95% confidence interval level. The calculated sample size was 3,466 older adults. Due to the possibility of subject absence or loss of data, the sample size was increased by 10%, resulting in a sample size of 3,812. However, the present study used data from the eighth TNOHS; thus, the data of 4,130 older adults were used.

The eighth TNOHS was conducted from June–August 2017. Data were collected by using interviews and clinical oral examinations by trained interviewers and trained dentists, respectively. For face and content validity, the standard forms for clinical oral examinations and a questionnaire was created and adjusted by experts in community oral health following the WHO oral health survey’s basic method [24], considering its appropriateness for the Thai cultural context, practicality, and time consumed during data collection. The questionnaire was tested with a group of older adults who were not the study sample. The results were re-evaluated by experts in community oral health and questions that were difficult to answer were excluded. The questionnaire and data collecting procedures were approved by the Bureau of Dental Health, Ministry of Public Health of Thailand.

Prior to performing interviews and clinical oral examinations, the interviewers and dentists attended a seminar, training and calibration exercises. The interviewers attended a seminar and training about the survey process, the questionnaire, and the appropriate way to interview individuals in this age group. Next, they made an agreement on standard adjustment with the Bureau of Dental Health of Thailand staff. All the examined dentists attended the seminar, training and practice sessions at Bang Len Hospital, Nakhon Pathom Province. The examination results from the examined dentist were calibrated with other examined dentists and the experts, after which an agreement was made on standard adjustment, which had to be 80% agreement or more and more than 0.8 on kappa coefficient for standard adjustment. This method was similar to that used in the seventh TNOHS [5].

### Ethical considerations

The protocol was approved by the Human Research Ethics Committee of the Faculty of Dentistry, Chulalongkorn University, Bangkok, Thailand (HREC-DCU 2019–002).

### Variables of interest

This study explored social backgrounds, DSU, chewing difficulty, and oral status data. The social backgrounds questionnaire consisted of questions on the following: (1) area of living (‘urban’ or ‘rural’); (2) gender (‘male’ or ‘female’); (3) age (60–64, 65–69 or 70–74); (4) social welfare (UCS, SSS or CSMBS); (5) Monthly income (‘ ≤ 15,000 baht’ or ‘ > 15,000 baht’); (6) highest education level (‘primary school’ or ‘above primary school’) and (7) independence of the elderly (‘completely independent’, ‘partially independent’ or ‘fully dependent’). The DSU questionnaire asked one question as follows: (1) Have you used dental services during the past year? (‘yes’ or ‘no’). The chewing difficulty questionnaire asked one question as follows: (1) Do you have any problems chewing food in daily life? (‘no problems’, ‘moderate chewing problems’ or ‘severe chewing problems’. The oral status data were collected by oral examination on the following topics: (1) dentition status; (2) posterior occlusal pairs; (3) oral dryness (‘yes’ or ‘no’) and (4) prosthesis status (‘removable prosthesis’ or ‘no removable prosthesis’).

### Statistical analysis

The data were entered into SPSS software twice to recheck for potential data entry errors. The data were analyzed by using the SPSS software package (Version 22.0, SPSS, Chicago, IL, USA). The significance level was set at 5%. Descriptive statistics were used to report social backgrounds, DSU, chewing difficulty, and oral status. The bivariate analysis, chi-square testing, was used to explore the associations between social backgrounds, DSU, oral status, and chewing difficulty. Dependent variables with *p* value of < 0.2 for their association with independent variables in the bivariate analysis were entered into the multiple logistic regression models.

## Results

The number of older adults who participated in this survey was 4,130. The participants’ social backgrounds and DSU data are shown in Table 1. Fifty-five percent lived in urban areas. Slightly more females (51.5%) participated than males. Older adults between 70 and 74 years old were the majority of age group participating (36.0%). The majority of older adults were entitled to USC (79.8%). Most of the older adults had income under 15,000 baht per month (90.9%) and primary school education or less (78.0%). Ninety-six percent of the older adults’ independence was completely independent. More than half of the older adults had not visited a dentist during the previous year (61.6%). The participants’ oral statuses are shown in Tables 2 and 3. More than half of the older adults had fewer than 20 teeth (53.9%). Approximately one-third of the older adults had 21–27 teeth. Only 10% of the older adults had 28 teeth or more (13.8%). The percentage of older adults who had fewer than four occlusal pairs was 60.9%, while nearly 30% had 4–7 occlusal pairs, and approximately 10% had eight occlusal pairs or more. The participants’ oral dryness condition and prosthesis statuses are shown in Table 3. Five percent of the older adults had oral dryness problem. Percentages of older adults who wore upper removable prostheses, lower removable prostheses, and upper and lower removable prostheses were 23.0%, 17.3% and 24.8%, respectively. Approximately 53% of the older adults had chewing difficulty.

**Table 1:** Associations between social backgrounds, dental service utilization (DSU), and chewing difficulty in Thai older adults (*n* = 4,130)

Variables	N (%)	% with Chewing difficulty
*Location*		
Urban	2,242 (54.3)	53.7
Rural	1,888 (45.7)	51.6^¶^
*Gender*		
Male	2,001 (48.5)	52.8
Female	2,129 (51.5)	52.7
*Age*		
60–64 yrs	1,383 (33.4)	52.1
65–69 yrs	1,262 (30.6)	50.4
70–74 yrs	1,485 (36.0)	55.3*
*Welfare*		
UCS	3,294 (79.8)	53.9
SSS	142 (3.4)	50.0
CSMBS	694 (16.8)	47.6**
*Income*		
≤ 15,000 baht	3,756 (90.9)	53.6
> 15,000 baht	374 (9.1)	43.6***
*Education*		
Primary school or lower	3,222 (78.0)	54.0
Middle school or higher	908 (22.0)	48.2**
*Elderly independence*		
Completely independent	3,961 (95.9)	52.5
Partially independent or Fully dependent	169 (4.1)	58.6^¶^
*DSU*		
Not utilized	2,543 (61.6)	50.1
Utilized	1,587 (38.4)	57.0***

***p < 0.001, **p < 0.01, *p < 0.05, ^¶^*p* < 0.2 (Chi-square test)

UCS, Universal Coverage Scheme; SSS, Social Security Scheme; CSMBS, Civil Servant Medical Benefit Scheme

**Table 2:** Associations between oral status and chewing difficulty in Thai older adults (*n* = 4,130)

Variables		N (%)	% with Chewing difficulty
*Number of teeth*			
20	< 20	2,228 (53.9)	53.6
	≥ 20	1,902 (46.1)	51.7
21	< 21	2,355 (57.0)	53.8
	≥ 21	1,775 (43.0)	51.4^¶^
22	< 22	2,482 (60.1)	53.6
	≥ 22	1,648 (39.9)	51.5^¶^
23	< 23	2,635 (63.8)	53.7
	≥ 23	1,495 (36.2)	51.0^¶^
24	< 24	2,818 (68.2)	53.4
	≥ 24	1,312 (31.8)	51.3
25	< 25	3,008 (72.8)	53.4
	≥ 25	1,122 (27.2)	51.1^¶^
26	< 26	3,205 (77.6)	53.6
	≥ 26	925 (22.4)	49.7^*^
27	< 27	3,383 (81.9)	53.6
	≥ 27	747 (18.1)	49.0^*^
28	< 28	3,559 (86.2)	53.6
	≥ 28	571 (13.8)	47.3^**^
*Occlusal Pairs*			
4	< 4	2,517 (60.9)	53.0
	≥ 4	1,613 (39.1)	52.3
5	< 5	2,843 (68.8)	53.3
	≥ 5	1,287 (31.2)	51.4
6	< 6	3,141 (76.1)	53.3
	≥ 6	989 (23.9)	51.0
7	< 7	3,470 (84.0)	53.3
	≥ 7	660 (16.0)	49.5^¶^
8	< 8	3,705 (89.7)	53.4
	≥ 8	425 (10.3)	47.3*

**p < 0.01, *p < 0.05, ^¶^*p* < 0.2 (chi-square test)

**Table 3:** Associations between oral dryness, prosthesis conditions and chewing difficulty in Thai older adults (*n* = 4,130)

Variables		N (%)	% with Chewing difficulty
*Xerostomia (mouth mirror stick)*			
	Yes	206 (5.0)	54.4
	No	3,924 (95.0)	52.7
*Prosthesis status*			
Upper	Fixed or none	3,179 (77.0)	52.3
	Removable	951 (23.0)	54.2
Lower	Fixed or none	3,416 (82.7)	52.3
	Removable	714 (17.3)	55.0^¶^
Upper and lower	Fixed or none	3,104 (75.2)	52.5
	Removable	1,026 (24.8)	54.5^¶^

^¶^*p* < 0.2 (chi-square test)

The bivariate analysis revealed significant associations between chewing difficulty and some social backgrounds and between DSU and some oral statuses (Tables 1, 2, 3). When comparing chewing difficulty in older adults with different ages, welfare, income and greater prevalence of chewing difficulty were found to be more prevalent in the group of older adults who were 70–74 years old than in other age groups with more in the UCS welfare group than in other welfare groups and more among those with low income than in the high income group. When comparing the chewing difficulty of older adults with difference in dental service utilization frequency, the group of older adults who had utilized dental services in the previous year had more prevalence of chewing difficulty than the group that had not utilized dental services. When comparing the chewing difficulty of older adults with different numbers of teeth and occlusal pairs, the group of elderly who had at least 27 teeth or at least eight occlusal pairs had less prevalence of chewing difficulty than the group of older adults who had fewer than 27 teeth or fewer than eight occlusal pairs (Table 4).

**Table 4:** Multiple logistic regression models for the associations of social backgrounds, dental service utilization (DSU), and oral status with chewing difficulty among Thai older adults (*n* = 4,130)

Variable	% with Chewing difficulty					
	Model 1 (95% CI)	Model 2.1 (95% CI)	Model 2.2 (95% CI)	Model 2.3 (95% CI)	Model 2.4 (95% CI)	Model 2.5 (95% CI)
*Location*						
Urban	1	1	1	1	1	1
Rural	0.93 (0.82, 1.05)	0.93 (0.82, 1.05)	0.93 (0.82, 1.05)	0.93 (0.82, 1.05)	0.93 (0.82, 1.05)	0.93 (0.82, 1.05)
*Age*						
60–64 yrs	1	1	1	1	1	1
65–69 yrs	0.93 (0.80, 1.08)	0.92 (0.79, 1.08)	0.92 (0.79, 1.08)	0.92 (0.79, 1.08)	0.92 (0.79, 1.08)	0.92 (0.79, 1.07)
70–74 yrs	1.13 (0.98, 1.32)	1.12 (0.96, 1.30)	1.12 (0.96, 1.30)	1.12 (0.96, 1.30)	1.12 (0.97, 1.30)	1.11 (0.96, 1.29)
*Welfare*						
UCS	1	1	1	1	1	1
SSS	0.92 (0.65, 1.30)	0.92 (0.65, 1.29)	0.92 (0.65, 1.29)	0.92 (0.65, 1.29)	0.91 (0.65, 1.29)	0.91 (0.64, 1.28)
CSMBS	0.86 (0.71, 1.04)	0.86 (0.71, 1.04)	0.86 (0.71, 1.04)	0.86 (0.72, 1.04)	0.86 (0.71, 1.04)	0.86 (0.71, 1.04)
*Education*						
Primary school or lower	1	1	1	1	1	1
Middle school or higher	0.87 (0.74, 1.03)	0.87 (0.74, 1.03)	0.87 (0.74, 1.03)	0.87 (0.74, 1.03)	0.87 (0.74, 1.03)	0.87 (0.74, 1.03)
*Income*						
≤ 15,000 baht	1	1	1	1	1	1
> 15,000 baht	0.72 (0.56, 0.92)*	0.72 (0.56, 0.92)**	0.72 (0.56, 0.92)**	0.72 (0.56, 0.92)**	0.72 (0.56, 0.92)**	0.72 (0.56, 0.92)**
*Elderly independence*						
Completely independent	1	1	1	1	1	1
Partially independent or Fully dependent	1.27 (0.93, 1.74)	1.32 (0.93, 1.74)	1.27 (0.93, 1.74)	1.27 (0.93, 1.74)	1.27 (0.93, 1.74)	1.27 (0.92, 1.74)
*DSU*						
Not utilized	1	1	1	1	1	1
Utilized	1.37 (1.20, 1.55)***	1.36 (1.20, 1.55)***	1.36 (1.20, 1.55)***	1.36 (1.20, 1.55)***	1.37 (1.20, 1.55)***	1.36 (1.20, 1.55)***
*Number of Teeth*						
< 21	–	1	–	–	–	–
≥ 21	–	0.93 (0.82, 1.05)	–	–	–	–
< 22	–	–	1	–	–	–
≥ 22	–	–	0.93 (0.82, 1.06)	–	–	–
< 23	–	–	–	1	–	–
≥ 23	–	–	–	0.92 (0.81, 1.05)	–	–
< 25	–	–	–	–	1	–
≥ 25	–	–	–	–	0.93 (0.81, 1.07)	–
< 26	–	–	–	–	–	1
≥ 26	–	–	–	–	–	0.87 (0.75, 1.01)

Variable	% with Chewing difficulty					
	Model 2.6 (95% CI)	Model 2.7 (95% CI)	Model 2.8 (95% CI)	Model 2.9 (95% CI)	Model 2.10 (95% CI)	Model 2.11 (95% CI)
*Location*						
Urban	1	1	1	1	1	1
Rural	0.93 (0.82, 1.06)	0.93 (0.82, 1.06)	0.93 (0.82, 1.06)	0.93 (0.82, 1.06)	0.93 (0.82, 1.06)	0.93 (0.82, 1.06)
*Age*						
60–64 yrs	1	1	1	1	1	1
65–69 yrs	0.92 (0.79, 1.07)	0.92 (0.78, 1.07)	0.92 (0.79, 1.08)	0.92 (0.79, 1.07)	0.93 (0.79, 1.08)	0.93 (0.79, 1.08)
70–74 yrs	1.11 (0.96, 1.29)	1.11 (0.95, 1.28)	1.12 (0.96, 1.30)	1.12 (0.96, 1.30)	1.13 (0.97, 1.31)	1.13 (0.97, 1.31)
*Welfare*						
UCS	1	1	1	1	1	1
SSS	0.91 (0.64, 1.28)	0.91 (0.64, 1.28)	0.91 (0.65, 1.29)	0.91 (0.64, 1.28)	0.92 (0.65, 1.29)	0.92 (0.65, 1.29)
CSMBS	0.86 (0.71, 1.04)	0.86 (0.71, 1.04)	0.86 (0.71, 1.04)	0.86 (0.71, 1.04)	0.86 (0.72, 1.04)	0.86 (0.71, 1.04)
*Education*						
Primary school or lower	1	1	1	1	1	1
Middle school or higher	0.87 (0.74, 1.03)	0.87 (0.74, 1.03)	0.87 (0.74, 1.03)	0.87 (0.74, 1.03)	0.87 (0.74, 1.03)	0.87 (0.74, 1.03)
*Income*						
≤ 15,000 baht	1	1	1	1	1	1
> 15,000 baht	0.72 (0.56, 0.92)**	0.72 (0.56, 0.92)**	0.72 (0.56, 0.92)**	0.72 (0.56, 0.92)*	0.72 (0.56, 0.92) *	0.72 (0.56, 0.92)**
*Elderly independence*						
Completely independent	1	1	1	1	1	1
Partially independent or Fully dependent	1.27 (0.93, 1.74)	1.27 (0.93, 1.74)	1.27 (0.93, 1.74)	1.27 (0.93, 1.74)	1.26 (0.92, 1.73)	1.26 (0.92, 1.73)
*DSU*						
Not utilized	1	1	1	1	1	1
Utilized	1.36 (1.20, 1.55)***	1.36 (1.20, 1.55)***	1.36 (1.20, 1.55)***	1.36 (1.20, 1.55)***	1.36 (1.20, 1.55)***	1.36 (1.20, 1.55)***
*Number of teeth*						
< 27	1					
≥ 27	0.84 (0.72, 0.99)*					
< 28		1				
≥ 28		0.78 (0.65, 0.94)*				
*Occlusal Pairs*						
< 7		–	1	–	–	–
≥ 7		–	0.87 (0.73, 1.03)	–	–	–
< 8		–	–	1	–	–
≥ 8		–	–	0.79 (0.65, 0.97)*	–	–
*Prosthesis Status: Lower*						
Fixed or none		–	–	–	1	–
Removable		–	–	–	1.06 (0.90, 1.26)	–
*Prosthesis Status: Upper and Lower*						
Fix or none		–	–	–	–	1
Removable		–	–	–	–	1.06 (0.92, 1.23)

*CI*, Confidence Interval; *UCS*, Universal Coverage Scheme; *SSS*, Social Security Scheme; *CSMBS*, Civil Servant Medical Benefit Scheme

Model 1: adjusted for social backgrounds and DSU; model 2: further adjusted for oral status.

^***^p < .001, ^**^p < .01, ^*^p < .05

## Discussion

The study provided evidence on the association of social backgrounds, DSU, and oral status with chewing difficulty. Our study found that social backgrounds, DSU and oral status were associated with chewing difficulty. The most commonly reported symptom in independently-living older adults who were 60 years and older was “uncomfortable to eat” [25].

For oral status, number of teeth and occlusal pairs are the leading cause of chewing difficulty. This study found that older adults who had at least 27 teeth or at least eight occlusal pairs were more likely to have less chewing difficulty. The reason for this finding might be that larger numbers of the teeth and occlusal pairs lead to better distribution and mastication. This finding is in accordance with a previous study showing an association between tooth loss and chewing difficulty. A systemic review by Naka and colleagues revealed that tooth loss is associated with chewing difficulty in older adults. Most studies have found that individuals with at least 20–21 teeth have satisfactory chewing ability [26]. Older adults with at least five posterior occlusal pairs have been found to have sufficient chewing ability [27, 28]. Our findings were inconsistent with the goal of the Ministry of Public Health of Thailand, the WHO, and the FDI. According to our findings, the goal to have at least 20–21 teeth and four occlusal pairs might not be sufficient to enhance the OHRQoL of older people. Thus, the goal of the minimum number of teeth and posterior occlusal pairs set by the Ministry of Public Health, the WHO, and the FDI should be higher. We suggest that policymakers increase the number of preventive plans for higher numbers of teeth and posterior teeth in young and working aged people, especially in the low-income group. Regarding the WHO’s priority action areas [29], we should promote more effective use of fluoride, more tobacco prevention, better oral health, general health and quality of life, as well as the oral health system. Healthy public policies, legislation, regulations, and fiscal measures can all be utilized to promote oral health either at local or national levels. For example, involving authorities should encourage tighter legislation on food labeling and food advertisement on products with support for the removal of taxes on fluoride toothpastes and toothbrushes [30].

An earlier study demonstrated the association between dry mouth and chewing problems [31]. However, this current study was unable to find this association, possibly due to differences in the methods of defining dry mouth. Locker [31] asked seven questions about dry mouth within four weeks preceding the study, while this study collected dry mouth data by clinical oral examination. Our study identified dry mouth by examining the mouth with a mouth mirror, if the mouth mirror stuck to the buccal mucosa or the tongue, the participant was indicated to have dry mouth.

Furthermore, the current study could not find an association between prosthesis status and chewing difficulty. On the contrary, other studies found relationships between prosthesis status and chewing difficulty. Adults with dentures tend to have more chewing problems than those with natural teeth [32]. In addition, the type of prosthesis used was associated with prevalence of chewing problems. The group of participants with removable dentures had more prevalence of chewing problems than the group with fixed dental prostheses [33]. However, this study did not find any differences in chewing difficulty between participants with removable dentures and participants without removable dentures, possibly due to the limited number of participants with fixed dental prostheses. Therefore, the participants with natural teeth and fixed dental prostheses were combined and classified as participants without removable dentures. Moreover, the differences in the results might have been due to differences in participant age. This study included only participants aged 60 years or over, while other studies included participants younger than 60 years [32, 33].

As expected, socioeconomic status was associated with chewing difficulty. This study found that older adults who had higher incomes had less chewing difficulty compared to their counterparts. This finding is consistent with another study conducted in Thailand in which Yiengprugsawan and colleagues demonstrated that adults who had income under 10,000 baht had more chewing difficulty than their counterparts [17]. Low socioeconomic status can affect general and oral health [34], while high income was associated with less chewing discomfort. Older adults in upper income quartiles were less likely to have chewing discomfort than older adults in lower income quartiles [35]. Apart from current socioeconomic status, childhood socioeconomic status was also associated with chewing difficulty later in life at 50 years or older [36]. After tooth extraction prosthesis treatment such as crowns, bridges and removal dentures are needed to close the gap between the teeth and maintain oral cavity function and esthetics. Unfortunately, people from low socioeconomic backgrounds may not be able to access prosthesis treatment as there is evidence that wealth index is associated with the number of non-replaced extracted teeth. The participants with lower income have more non-replaced extracted teeth than the participants with higher income [34]. In addition, lower income was associated with more edentulousness. Among dentate participants, however, lower income was associated with more oral impacts, including food eating difficulty [37].

An association between age, education, and chewing difficulty was reported earlier [17]; however, the present study did not find any associations between age, education, and chewing difficulty, possibly due to differences in subject age. The range of the subjects’ age was 60–74 years. In another study, the participants were in the wider range of 15–87 years. Moreover, education was categorized differently. In a previous study, education was divided into three groups: high school or less, diploma, and university degree [17], while this study divided education into two groups: ‘primary school or lower’ and ‘middle school or higher’.

This study could not find an association between social welfare and chewing difficulty. This finding was inconsistent with the findings of a previous study revealing that welfare dental intervention improved OHRQoL and made it comfortable to eat [38]. The dissimilarity between the results of these studies may be due to differences in social welfare schemes. Our study included three social welfare schemes, namely the Civil Servant Medical Benefit Scheme, the Social Security Scheme and the Universal Coverage Scheme. Furthermore, the participants in each group received different benefits, while other study’s participants had the same benefits.

For the association between DSU and chewing difficulty, we found that older adults who utilized dental services were associated with more chewing difficulty. This finding was comparable to the earlier study in Canada. The study demonstrated that dental problems were associated with DSU [19]. The participants in the Canadian study were 1,537 Chinese Canadians aged 55 years and older. The study showed that, among older adult Chinese immigrants in Canada, 52% of the study participants had not used dental services in the previous year and nearly 41% had dental problems. The study found that the immigrants who had dental problems were more likely to use dental services.

One limitation of this study was the number of only three answers for the chewing difficulty question. Another limitation of this study was the study design, which was a cross-sectional study. Thus, we were not able to determine changes in chewing difficulty over time. Additional longitudinal studies and time-series data are required to test the validity of these factors. The questions in the questionnaire were an additional limitation of this study, despite the fact that this questionnaire was created and re-evaluated by the experts. The study used secondary data from the eighth TNOHS; thus, the questions were limited to the questions from the survey.

Despite the limitations, there were several strengths to this study. Firstly, it was conducted on a national scale with over 4,000 Thai older adults representing the Thai older adult population well in terms of social backgrounds, DSU, oral status and chewing difficulty. Moreover, due to the large number of subjects this study had power of approximately 90%; higher power decreases the possibility of Type II errors. The standardized data collection method in this study was created and adjusted by experts in community oral health according to the basic method of the WHO oral health survey. The questionnaire was tested and re-evaluated by experts in the field and approved by the Bureau of Dental Health, Ministry of Public Health, Thailand. The interviewers and examiners received calibration training and made an agreement on standard adjustment by the Bureau of Dental Health. Finally, we analyzed our results by using multiple logistic regression, adjusting for social backgrounds, DSU, and oral status with chewing difficulty. This method avoids confounding effects during the analysis and allows simultaneous multiple comparisons.

## Conclusions

Chewing difficulty was associated with number of teeth and posterior occlusal pairs. Thai older adults who had at least 27 teeth or at least eight posterior occlusal pairs were more likely to have less chewing difficulty than their counterparts, while no association between prosthesis status and chewing difficulty was found after adjusting for confounders. Dental service utilization was associated with chewing difficulty. Thai older adults who utilized dental service had more prevalence of chewing difficulty than their counterparts. Income was the only social backgrounds variable associated with chewing difficulty, whereby Thai older adults with income exceeding 15,000 baht per month had lower prevalence of chewing difficulty than their counterparts.

## Data Availability

The datasets used and/or analyzed during the current study are available from the corresponding author on reasonable request. The data are not publicly available due to privacy or ethical restrictions.

## Abbreviations

WHO: World Health Organization
FDI: World Dental Federation
TNOHS: Thai National Oral Health Survey
TMJs: Temporomandibular joints
OHRQoL: Oral Health Related Quality of Life
ADLs: Activities of Daily Living
CSMBS: Civil Servant Medical Benefit Scheme
UCS: Universal Coverage Scheme
SSS: Social Security Scheme
DSU: Dental Service Utilization
OHIP: Oral Health Impact Profile
